# Phytochemical study of the headspace volatile organic compounds of fresh algae and seagrass from the Adriatic Sea (single point collection)

**DOI:** 10.1371/journal.pone.0196462

**Published:** 2018-05-08

**Authors:** Igor Jerković, Zvonimir Marijanović, Marin Roje, Piotr M. Kuś, Stela Jokić, Rozelinda Čož-Rakovac

**Affiliations:** 1 Department of Organic Chemistry, Faculty of Chemistry and Technology, University of Split, HR Split, Croatia; 2 Department of Food Technology and Biotechnology, Faculty of Chemistry and Technology, University of Split, HR Split, Croatia; 3 Division of Organic Chemistry and Biochemistry, Ruđer Bošković Institute, HR Zagreb, Croatia; 4 Department of Pharmacognosy, Wrocław Medical University, Wrocław, Poland; 5 Department of Process Engineering, Faculty of Food Technology, Josip Juraj Strossmayer University of Osijek, HR Osijek, Croatia; 6 Laboratory for Aquaculture Biotechnology, Ruđer Bošković Institute, HR Zagreb, Croatia; Tallinn University of Technology, ESTONIA

## Abstract

Performed phytochemical study contributes to the knowledge of volatile organic compounds (VOCs) of *Halopteris filicina* (Grateloup) Kützing, *Dictyota dichotoma* (Hudson) J. V. Lamouroux, *Posidonia oceanica* (L.) Delile and *Flabellia petiolata* (Turra) Nizamuddin from the Adriatic Sea (single point collection). VOCs were investigated by headspace solid-phase microextraction (HS-SPME) and analysed by gas chromatography and mass spectrometry (GC-MS/FID). *H*. *filicina* headspace contained dimethyl sulfide (DMS; 12.8%), C_8_-compounds (e.g. fucoserratene (**I**; 9.5%)), benzaldehyde (**II**; 8.7%), alkane C_17_, dictyopterene D and C (**III**, **IV**), tribromomethane (**V**), 1-iodopentane, others. *F*. *petiolata* headspace was characterized by DMS (22.2%), 6-methylhept-5-en-2-one (9.5%), C_17_ (9.1%), **II** (6.5%), compounds **I**-**V**. DMS (59.3%), C_15_ (14.5%), C_17_ (7.2%) and C_19_ (6.3%) dominated in *P*. *oceanica* headspace. Sesquiterpenes were found in *D*. *dichotoma*, predominantly germacrene D (28.3%) followed by other cadinenyl (abundant), muurolenyl and amorphenyl structures. Determined VOCs may be significant for chemosystematics and chemical communications in marine ecosystem.

## Introduction

Volatile organic compounds (VOCs) are low-molecular compounds with low to moderate hydrophilicity that can not only be dissolved in water, but also dissipate into the gas phase at air-water interfaces. Prior to 1966, only one volatile substance was identified from wet, undecomposed seaweed and described in the literature as dimethyl sulfide (DMS). Afterwards, the number of identified marine plant VOCs has been constantly growing. In 1976, VOCs from marine algae were reviewed by Moore [1] including non-isoprenoid C_11_-compounds, acyclic undecapolyenes, less volatile organosulfur compounds, bromine- and iodine-containing haloforms, halogenated compounds, others. The research on the algae VOCs has been continued by different teams [2–4]. The production and role of volatile halogenated compounds from marine algae were reviewed in 2011 [5]. Recently, more research papers on this topic appeared (e.g. [6,7]). VOCs biogeneration by microalgae, their occurrence, behaviour, ecological implications and industrial applications were described in 2016 [8]. The volatile metabolites emission by *in vivo* microalgae were reviewed in 2017 [9]. Tricyclic sesquiterpenes from marine origin were systematically presented in 2017 [10].

The common VOCs released by terrestrial plants are: ‘‘green leaf volatiles” (GLVs) which consist almost exclusively of C_6_ aldehydes and alcohols [11], other aliphatic compounds, monoterpenes, sesquiterpenes, phenylpropane derivatives, others [12]. This is in remarkable contrast to marine VOCs that show much higher structural diversity [13]: e.g. only among marine sesquiterpenes 54 different skeletal types of tricyclic sesquiterpenes were found [10]. In terrestrial ecosystems VOCs are very important group of infochemicals (e.g. plant-herbivore interactions, pheromones). Similarly, VOCs of marine organisms can have multiple functions in ecosystem in intraspecific (pheromones) and interspecific (kairomones) communication and in activated defences [14]. In addition, these compounds (usually present in the extracts) may exhibit other biological activities, e.g. antibacterial, cytotoxic and antitumor [10]. Different methods have been used to investigate VOCs from marine plants such as liquid-liquid extraction (LLE), hydrodistillation (HD), simultaneous distillation-extraction (SDE), vacuum-SDE (V-SDE), static and dynamic headspace extraction techniques (SHE and DHE) as well as headspace solid-phase microextraction (HS-SPME).

However, limited number of studies have been carried out on marine algae and seagrass headspace VOCs in general [6,7,15,16]. In addition, to the best of our knowledge, there is only one paper [17] about VOCs (obtained by SDE) of the algae from the Adriatic Sea reporting the chemical composition of *Padina pavonia* (L.) Gaill. Considering limited data available on the chemical composition of marine plants found in the Adriatic Sea, the focus of this study was on 4 different samples collected from the same location (single point collection): 3 seaweeds (marine macroalgae) - 2 brown algae (*Halopteris filicina* (Grateloup) Kützing, *Dictyota dichotoma* (Hudson) J. V. Lamouroux) and 1 green alga (*Flabellia petiolata* (Turra) Nizamuddin) as well as 1 seagrass (*Posidonia oceanica* (L.) Delile). *F*. *petiolata* has been commonly found in Mediterranean basin [18], often in association with other algae (e.g. *Dictyopteris* spp., *Dictyota* spp., *Dilophus* spp.). It is one of the main components of the phytocoenoses associated with the endemic and endangered seagrass *P*. *oceanica* [19,20]. The specific goals of present phytochemical study are: 1) to determine and unlock the headspace VOCs composition of targeted marine plants by headspace solid-phase microextraction (HS-SPME)/gas chromatography and mass spectrometry (GC-MS/FID)—first time report; 2) to compare the chemical biodiversity of found VOCs among the samples with other marine plants with same, similar or related constituents; 3) to discuss possible biosynthetic origin of identified VOCs from the literature data.

## Materials and methods

### The marine plant samples

We state clearly that no specific permissions were required for the location and collection. There is no need to issue the permission to collect the samples used in this work because the sample were not taken from national park or protected area, but from the public place–sea. The Ministry approved this type of research by approving our research projects: Croatian Science Foundation under the project HRZZ-IP-11-2013-8547 (Research of Natural Products and Flavours: Chemical Fingerprinting and Unlocking the Potential) and by the Croatian Ministry of Science and Education (MZO grant no. KK.01.1.1.01.0002 –The Scientific Center of Excellence for Marine Bioprospecting-BioProCro). We confirm that the field study did not involve endangered or protected species.

The samples of 2 brown algae (*Halopteris filicina* (Grateloup) Kützing, *Dictyota dichotoma* (Hudson) J. V. Lamouroux) and 1 green alga (*Flabellia petiolata* (Turra) Nizamuddin) as well as 1 seagrass (*Posidonia oceanica* (L.) Delile) were collected in the middle part of the Adriatic Sea, close to Mala Smokvica island, in July 2017 at the same geographic location (43^o^31'04.4"N, 15^o^56'32.6"E). Single point sample collection provided representative robust samples for first time chemical profiling. The samples were collected at 11 a.m. under sunny conditions—constantly illuminated, and area receive direct sunlight for >80% of the time from sunrise to sunset. During the samples collection there was no wind and the sea temperature was 22°C. The algae were collected from depth of 0.5–3 m and the sea was collected from the same depth (directly into the plastic bag where collected algae were placed). The samples were separately collected and separately placed in air tight plastic bags containing seawater and were immediately transported to the laboratory. The samples were kept in dark at 4°C until the extractions that were performed within 24 h after the collection. Before HS-SPME, each sample was taken out of the bag, cut into small pieces, and the excess of seawater was removed by placing it between filter paper layers for 2 min (the seawater was not removed completely).

### Headspace solid-phase microextraction (HS-SPME)

The headspace extraction was performed with a manual SPME holder using polydimethylsiloxane/divinylbenzene (PDMS/DVB) fibre obtained from Supelco Co. (Bellefonte, PA, USA). The fibre was conditioned prior to the extraction according to the instructions by Supelco Co. For HS-SPME, previously prepared samples (1 g) were placed separately in 5 mL glass vials and hermetically sealed with PTFE/silicone septa. The vials were maintained in a water bath at 60 ^o^C during equilibration (15 min) and HS-SPME (45 min). After sampling, the SPME fibre was withdrawn into the needle, removed from the vial, and inserted into the injector (250 ^o^C) of the GC-FID and GC-MS for 6 min where the extracted volatiles were thermally desorbed directly to the GC column. HS-SPME was done in triplicate for each sample.

### Gas chromatography and mass spectrometry (GC-FID and GC-MS) analyses

The GC-FID analyses were carried out with an Agilent Technologies (Palo Alto, CA, USA) gas chromatograph model 7890A equipped with a flame ionization detector (FID) and a HP-5MS capillary column (5% phenyl-methylpolysiloxane, Agilent J and W). The GC conditions were similar to those described previously [21]. In brief, the oven temp. was programmed isothermal at 70 ^o^C for 2 min, increasing from 70–200 ^o^C at 3 ^o^C min^-1^, and held isothermally at 200 ^o^C for 15 min; carrier gas was helium (He 1.0 mL min^-1^). The GC-MS analyses were performed using an Agilent Technologies (Palo Alto, CA, USA) gas chromatograph model 7820A equipped with a mass selective detector (MSD) model 5977E (Agilent Technologies) and a HP-5MS capillary column, under the same conditions as for the GC-FID analysis. The MSD (EI mode) was operated at 70 eV, and the mass range was 30–300 amu.

The identification of VOCs was based on the comparison of their retention indices (RI), determined relative to the retention times of *n*-alkanes (C_9_-C_25_), with those reported in the literature [22] and their mass spectra with the spectra listed in Wiley 9 (Wiley, New York, NY, USA) and NIST 14 (D-Gaithersburg) mass spectral libraries. The percentage composition of the samples was computed from the GC peak areas using the normalization method (without correction factors). The average component percentages in Table 1 were calculated from GC-FID and GC-MS analyses (triplicates).

**Table 1:** VOCs composition determined by headspace solid-phase microextraction (HS-SPME).

****No.****	Compound	Molecular formula	Compound class	RI	Area percentage (%)			
					1	2	3	4
1.	Ethanol	C_2_H_5_O	Alcohol	< 900	1.2	0.6	5.8	1.2
2.	Methylthiomethane (Dimethyl sulfide)	C_2_H_6_S	Organosulfur compound	< 900	12.8	22.2	59.3	1.0
3.	3-Methylbutanal	C_5_H_10_O	Aldehyde	< 900	0.9	1.0	0.0	0.0
4.	2-Methylbutanal	C_5_H_10_O	Aldehyde	< 900	0.5	0.0	0.0	0.0
5.	Benzene	C_6_H_6_	Aromatic compound	< 900	0.0	0.0	0.0	0.1
6.	Pent-1-en-3-one	C_5_H_8_O	Unsaturated ketone	< 900	2.6	2.9	0.0	0.2
7.	Pentanal	C_5_H_10_O	Aldehyde	< 900	0.0	1.6	0.0	0.1
8.	(2*E*)-Pent-2-enal	C_5_H_8_O	Aldehyde	< 900	0.0	0.0	0.0	0.1
9.	Dimethyl disulfide	C_2_H_6_S_2_	Organosulfur compound	< 900	0.0	0.0	0.0	0.1
10.	3-Methylbut-2-enal	C_5_H_8_O	Aldehyde	< 900	1.5	1.6	0.0	0.1
11.	(2*Z*)-Pent-2-en-1-ol	C_5_H_10_O	Unsaturated alcohol	< 900	0.9	0.6	0.0	0.1
12.	Methylbenzene	C_7_H_8_	Aromatic compound	< 900	0.0	0.0	0.0	0.1
13.	Hexanal	C_6_H_12_O	Aldehyde	< 900	4.7	1.5	0.2	0.3
14.	(3*E*)-Octa-1,3-diene	C_8_H_14_	Alkene	< 900	2.3	0.7	0.0	0.7
15.	(2*E*)-Hex-2-enal	C_6_H_10_O	Unsaturated aldehyde	< 900	0.5	0.2	0.0	0.0
16.	Hexan-1-ol	C_6_H_14_O	Alcohol	< 900	2.4	0.0	0.0	0.0
17.	(3*Z*,5*E*)-Octat-1,3,5-triene (Fucoserraten)	C_8_H_12_	Alkene	< 900	9.5	3.2	0.0	1.6
18.	Tribromomethane	CHBr_3_	Polyhalo-methane	< 900	2.1	1.8	0.0	0.0
19.	Heptanal	C_7_H_14_O	Aldehyde	903	0.3	0.5	0.0	0.1
20.	1-Iodopentane*	C_5_H_11_I	Organoiodine	929	0.8	0.0	0.0	0.0
21.	(2*E*,4*E*,6*E*)-Octa-2,4,6-triene*	C_8_H_12_	Alkene	941	0.2	0.0	0.0	0.1
22.	α-Pinene	C_10_H_16_	Monoterpene	942	0.0	0.0	0.1	0.0
23.	(2*Z*)-Hept-2-enal	C_7_H_12_O	Unsaturated aldehyde	961	0.3	0.6	0.0	0.1
24.	Benzaldehyde	C_7_H_6_O	Aldehyde	968	8.7	6.5	0.2	0.5
25.	Dimethyl trisulfide	C_2_H_6_S_3_	Organosulfur compound	978	0.0	0.0	0.0	0.1
26.	(5*Z*)-Octa-1,5-dien- 3-ol	C_8_H_14_O	Unsaturated alcohol	978	0.7	0.0	0.0	0.0
27.	Oct-1-en-3-one	C_8_H_14_O	Unsaturated ketone	981	0.5	1.1	0.0	0.1
28.	Oct-1-en-3-ol	C_8_H_16_O	Unsaturated alcohol	983	1.2	2.5	0.0	0.2
29.	Octan-3-one	C_8_H_16_O	Ketone	987	4.1	0.0	0.0	0.0
30.	6-Methylhept- 5-en-2-one	C_8_H_14_O	Unsaturated ketone	989	0.0	9.5	0.0	0.7
31.	Octan-2-one	C_8_H_16_O	Ketone	993	0.0	1.6	0.0	0.0
32.	2-Pentylfuran	C_9_H_14_O	Furan derivative	994	0.7	0.0	0.2	0.0
33.	(2*E*,4*Z*)-Hepta-2,4-dienal	C_7_H_10_O	Unsaturated aldehyde	999	2.4	0.7	0.0	0.2
34.	Octanal	C_8_H_16_O	Aldehyde	1004	4.7	0.5	0.0	0.0
35.	(2*E*,4*E*)-Hepta-2,4-dienal	C_7_H_10_O	Unsaturated aldehyde	1014	0.7	0.8	0.0	0.0
36.	1,8-Cineole	C_10_H_18_O	Monoterpene	1037	0.0	0.6	0.0	0.1
37.	Phenylacetladehyde	C_8_H_8_O	Aromatic aldehyde	1050	0.9	0.7	0.1	0.0
38.	(2*E*)-Oct-2-enal	C_8_H_14_O	Unsaturated aldehyde	1062	0.7	0.5	0.0	0.1
39.	Octan-1-ol	C_8_H_18_O	Alcohol	1077	5.1	0.0	0.0	0.3
40.	Nonanal	C_9_H_18_O	Aldehyde	1105	0.4	0.3	0.0	0.1
41.	6-[(1*Z*)-1-Butenyl]-1,4-cycloheptadiene (Dictyopterene D)	C_11_H_16_	Alkene	1156	1.9	7.4	0.0	0.0
42.	6-Butyl-1,4-cycloheptadiene (Dictyopterene C)	C_11_H_18_	Alkene		0.7	0.7	0.0	0.0
43.	Decanal	C_10_H_20_O	Aldehyde	1207	0.2	0.0	0.0	0.0
44.	β-Cyclocitral	C_10_H_16_O	C_13_-Norisoprenoid	1223	0.5	0.8	0.0	0.0
45.	Decan-1-ol	C_10_H_22_O	Alcohol	1279	0.0	0.0	0.0	0.1
46.	(2*E*,4*Z*)-Deca-2,4-dienal	C_10_H_1_6O	Unsaturated aldehyde	1295	0.5	0.0	0.0	0.0
47.	(2*E*,4*E*)-Deca-2,4-dienal	C_10_H_1_6O	Unsaturated aldehyde	1318	0.4	0.0	0.0	0.0
48.	α-Cubebene	C_15_H_24_	Sesquiterpene	1352	0.0	0.0	0.0	1.5
49.	Cycloisosativene	C_15_H_24_	Sesquiterpene	1367	0.0	0.0	0.0	0.5
50.	α-Ylangene	C_15_H_24_	Sesquiterpene	1372	0.0	0.0	0.0	0.3
51.	α-Copaene	C_15_H_24_	Sesquiterpene	1377	0.0	0.0	0.0	0.7
52.	β-Bourbonene	C_15_H_24_	Sesquiterpene	1386	0.0	0.0	0.0	5.1
53.	β-Cubebene	C_15_H_24_	Sesquiterpene	1390	0.0	0.0	0.0	0.8
54.	Tetradec-1-ene	C_14_H_28_	Alkene	1393	0.0	1.4	0.0	0.0
55.	Tetradecane	C_14_H_30_	Alkane	1400	0.0	0.0	0.6	0.0
56.	Dodecanal	C_12_H_24_O	Aldehyde	1409	0.3	0.0	0.0	0.0
57.	α-Ionone	C_13_H_20_O	C_13_-Norisoprenoid	1429	0.4	0.9	0.0	0.0
58.	Aromadendrene	C_15_H_24_	Sesquiterpene	1440	0.0	0.0	0.0	0.3
59.	(*E*)-β-farnesene	C_15_H_24_	Sesquiterpene	1459	0.0	0.0	0.0	1.0
60.	γ-Muurolene	C_15_H_24_	Sesquiterpene	1465	0.0	0.0	0.0	2.1
61.	β-Cadinene	C_15_H_24_	Sesquiterpene	1474	0.0	0.0	0.0	2.8
62.	α-Amorphene	C_15_H_24_	Sesquiterpene	1478	0.0	0.0	0.0	3.5
63.	Dodecan-1-ol	C_12_H_26_O	Alcohol	1479	0.4	0.0	0.0	0.0
64.	Germacrene D	C_15_H_24_	Sesquiterpene	1481	0.0	0.0	0.0	28.3
65.	Ar-curcumene	C_15_H_22_	Sesquiterpene	1484	0.7	0.0	0.0	0.9
66.	β-Ionone	C_13_H_20_O	C_13_-Norisoprenoid	1487	1.2	0.7	0.0	0.0
67.	Pentadec-1-ene	C_15_H_30_	Alkene	1493	3.3	3.2	0.0	0.0
68.	Bicyclogermacrene	C_15_H_24_	Sesquiterpene	1496	0.0	0.0	0.0	4.7
69.	Epizonarene	C_15_H_24_	Sesquiterpene	1498	0.0	0.0	0.0	4.3
70.	α-Muurolene	C_15_H_24_	Sesquiterpene	1499	0.0	0.0	0.0	2.2
71.	Pentadecane	C_15_H_32_	Alkane	1500	2.6	1.4	14.5	0.0
72.	δ-Selinene	C_15_H_24_	Sesquiterpene	1507	0.0	0.0	0.0	2.7
73.	Tridecanal	C_13_H_26_O	Aldehyde	1511	0.5	0.0	0.2	0.0
74.	γ-Cadinene	C_15_H_24_	Sesquiterpene	1515	0.0	0.0	0.0	3.4
75.	δ-Cadinene	C_15_H_24_	Sesquiterpene	1525	0.0	0.0	0.0	8.3
76.	*trans*-γ-Bisabolene	C_15_H_24_	Sesquiterpene	1533	0.3	0.0	0.0	0.0
77.	*trans*-Cadina-1,4-diene	C_15_H_24_	Sesquiterpene	1534	0.0	0.0	0.0	1.2
78.	α-Cadinene	C_15_H_24_	Sesquiterpene	1539	0.0	0.0	0.0	0.9
79.	α-Calacorene	C_15_H_24_	Sesquiterpene	1545	0.0	0.0	0.0	1.3
80.	Germacrene B	C_15_H_24_	Sesquiterpene	1555	0.3	0.0	0.0	3.6
81.	Dendrolasin	C_15_H_22_O	Furan derivative	1575	0.3	1.2	0.0	0.0
82.	Hexadecane	C_16_H_34_	Alkane	1600	0.0	0.0	0.4	0.0
83.	α-Cadinol	C_15_H_26_O	Sesquiterpene	1645	0.0	0.0	0.0	0.2
84.	τ-Muurolol	C_15_H_26_O	Sesquiterpene	1657	0.0	0.0	0.0	0.1
85.	(8*E*)-Heptadec-8-ene	C_17_H_34_	Alkene	1679	0.7	1.6	0.1	0.0
86.	Heptadec-1-ene	C_17_H_34_	Alkene	1693	1.1	0.6	0.0	0.0
87.	Heptadecane	C_17_H_36_	Alkane	1700	4.0	9.1	7.2	0.1
88.	Octadecanal	C_18_H_36_O	Aldehyde	1715	0.3	0.0	1.5	0.0
89.	Octadecane	C_18_H_38_	Alkane	1800	0.0	0.0	0.6	0.0
90.	Nonadecane	C_19_H_40_	Alkane	1900	0.0	0.0	6.3	0.1
91.	Heneicosane	C_21_H_44_	Alkane	2100	0.0	0.0	0.6	0.0
Total identified (%)					95.0	92.8	97.9	89.9

The symbols in the table are: **1 –***H*. *filicina*; **2** –*F*. *petiolata*; **3** –*P*. *oceanica*; **4** –*D*. *dichotoma*; RI–retention indices relative to C_9_-C_25_ alkanes

*—tentatively identified

## Results and discussion

Headspace solid-phase microextraction (HS-SPME) with the fibre polydimethylsiloxane/ divinylbenzene (PDMS/DVB) was successfully applied first time to investigate VOCs from four fresh marine plants (*Halopteris filicina* (Grateloup) Kützing, *Dictyota dichotoma* (Hudson) J. V. Lamouroux, *Posidonia oceanica* (L.) Delile and *Flabellia petiolata* (Turra) Nizamuddin) that were collected from the Adriatic Sea from the same location. Single point sample collection provided representative robust samples for first time chemical profiling. Representative chemical profiles of their headspace composition were obtained without the application of heat that can lead to the artefacts (as in other methods for VOCs isolation e.g. hydrodistillation). The first insight into the results (Table 1) reveals striking differences among the obtained chemical profiles. As was expected, significant contrast with common VOCs released by terrestrial plants can also be immediately noticed. The results are further discussed in separate subtitles for each seaweed or seagrass and for possible biosynthetic origin of identified VOCs.

### *Halopteris filicina* (Grateloup) Kützing headspace VOCs

*Halopteris filicina* (Grateloup) Kützing is a greenish-brown, fern-like seaweed that belongs to the family Stypocaulaceae and it has been usually found in the Mediterranean and warm seas. Preliminary phytochemical screening of its crude extracts revealed the presence of alkaloids, saponins, flavonoids and terpenes [23]. Free amino acids, amino sulfonic acids, sugars and sterols have been examined and quantitatively determined [24], but there is no report on its volatile constituents. Its methanolic extract showed inhibition against *Staphylococcus aureus*, *Staphylococcus epidermidis*, *Bacillus subtilis*, *Bacillus* spp., *Salmonella typhi*, and *Escherichia coli* [23].

The headspace VOCs composition of *H*. *filicina* is reported in Table 1. Dimethyl sulfide (DMS) was the most abundant (12.8%). DMS serves as a chemoattractant for phytoplankton, bacteria, zooplankton, fish, and sea birds [25,26]. Tribromomethane (2.1%) was found and it can be classified as polyhalomethane. The VOCs of *Ascophyllum nodosum* contained tribrommethane [27]. One organoiodine was identified: 1-iodopentane (0.8%). *Asparagopsis taxiformis*, *Asparagopsis armata* and *Falkenbergia rufolanosa* synthesized more than 100 different organoiodines including 1-iodopentane [28]. It has also been demonstrated that the volatile halogenated compounds are involved in the defence mechanism (allelopathy) of the algae [29] as one of several types of allelochemicals. They are probably important in the global cycling of gaseous organohalogen species [30]. The most widespread were aliphatic compounds, particularly C_8_-compounds (total 9 compounds, Table 1) such as: octan-1-ol (5.1%), octanal (4.7%), octan-3-one (4.1%), (3*E*)-octa-1,3-diene (2.3%), (3*Z*,5*E*)-octat-1,3,5-riene (9.5%), or oct-1-en-3-ol (1.2%). Two typical marine algae C_11_-hydrocarbons were also present: dictyopterene D (1.9%) and dictyopterene C (0.7%). Most of the chemical signals (specific volatile pheromones) in various genera of macroalgae are unsaturated, acyclic and/or alicyclic C_11_-hydrocarbons of different ring size and different degrees of unsaturation which are active at picomolar concentrations [14,31]. C_8_-Hydrocarbon fucoserratene ((3*Z*,5*E*)-octa-1,3,5-triene) is also used for intraspecific chemical communication (pheromone) in Fucales [14,32]. Another group of abundant aliphatic compounds were higher acyclic hydrocarbons such as heptadecane (4.0%), pentadecane (2.6%) or pentadec-1-ene (3.3%). Pentadecane was most typical in *Ulva* spp. and two species of *Enterornorpha*. Unbranched alkanes were identified in *Ulva rigida* with heptadecane predominance [33]. Three C_13_-norisoprenoids were detected: α-ionone (0.4%), β-ionone (1.2%) and β-cyclocitral (0.5%). C_13_-norisoprenoids, such as the potent flavour compound β-ionone, are produced by a diversity of algae taxa, for example *Ulothrix fimbriata* and *Porphyra tenera* [14]. Two phenypropane derivatives were found (Table 1): benzaldehyde (8.7%) and phenylacetladehyde (0.9%). Benzaldehyde was predominant in seaweed *Monostroma nitidum* [6].

Previous research applying GC-MS on *H*. *filicina* reported the amounts of fatty acids (evaluated as the sum of all fatty acids (originally present and those resulting from the alkaline hydrolysis) by GC-MS after derivatization with BF_3_ and purification) that ranged from 74 to 1897 mg kg^-1^, dry weight [34]. *H*. *filicina* significantly differs from the other species as it shows a much smaller fatty acids total content, although it contains important acids (e.g. hexadecanoic acid (C16:0), octadecanoic acid (C18:0), or eicosapentaenoic acid (EPA, C20:5 ω-3)). In addition, valuable compounds in macroalgae extracts were determined [35] by GC-MS after derivatisation with trimethylsilyltrifluoroacetamide (MSTFA). *H filicina* extract contained proline, mannitol, hexadecanoic acid, octadecanoic acid, cholestanol and fucosterol. Sterols were identified by comparison of GC retention data of their TMSi ethers with those of authentic compounds and by GC-MS of their acetates [24]. The sterol fraction contained fucosterol, cholesterol and 24-methylenecholesterol; minute amounts of 22-dehydrocholesterol and 24-methyl-cholesta-5,22-dien-3β-ol.

### *Flabellia petiolata* (Turra) Nizamuddin headspace VOCs

*Flabellia petiolata* (Turra) Nizamuddin is a green alga that belongs to Udoteaceae family (Chlorophyta, Bryopsidales) commonly found in the Mediterranean basin [36]. Compared to many other green algae, *F*. *petiolata* appears to be a particularly interesting species, since antibacterial, antiviral, antimitotic, antifungal and cytotoxic activities of its raw extract have been observed [37]. Selected constituents from *F*. *petiolata* from Turkey were determined [38] such as: the average cellulose content (18.86±0.69%), the average crude protein content (22.45±0.62%) and the crude fat content (1.08±0.24%). There were no available data on its VOCs composition.

DMS, a highly-volatile organosulfur compound was quite abundant in the headspace of *F*. *petiolata* (22.2%; Table 1) and in general serves to the algae as a chemoattractant [25,26]. Since DMS distribution in the water column does not correlate well with phytoplankton biomass [39], it led to suggestions that only certain groups of marine algae (e.g. *Emiliania huxleyi*) produce significant amounts of DMS. Polyhalomethane tribromomethane was present with low percentage (1.8%). Variety of aliphatic compounds were found in the headspace (Table 1). Low-molecular aliphatic compounds up to 7 carbons, particularly ketones and aldehydes, were quite widespread, e.g. 3-methylbutanal (1.0%), pentanal (1.6%), 3-methylbut-2-enal (1.6%), hexanal (1.5%), or pent-1-en-3-one (2.9%). In distinction from lipid-derived low-molecular aliphatic compounds, 3-methylbutanal is derived from leucine [16] and it is generally associated with heated products; however fresh *P*. *petiolata* was used for this research. It is frequently found in many sea products, such as crayfish or lobster as well as in red algae *Palmaria palmate* [14,38]. 6-Methylhept-5-en-2-one (9.5%) was the most abundant ketone and previously it was found in relatively high amount (along with 6-methylheptan-2-one) in V-SDE extract of the green alga *Capsosiphon fulvescens* [40]. Seven C_8_-compounds were identified with major representatives (3*Z*,5*E*)-octat-1,3,5-riene (fucoserratene; 3.2%), oct-1-en-3-ol (2.5%) and oct-1-en-3-one (1.1%). Identified dictyopterene D (7.4%) and dictyopterene C (0.7%) belong to C_11_-hydrocarbons (volatile pheromones). Heptadecane (9.1%) was the most abundant among alkanes and it was already observed to be one of the most common hydrocarbons in marine algae, detected in relevant amount (26.38%) in *Hypnea cornuta* [33]. C_13_-norisoprenoids were represented by α-ionone (0.9%), β-ionone (0.7%) and β-cyclocitral (0.8%). β-Ionone and β-cyclocitral were found previously among the major volatile compounds of the essential oil isolated from *Capsosiphon fulvescens* [40] by V-SDE. Benzaldehyde was the most abundant (6.5%) among phenylpropane derivatives. Only one monoterpene was found in low percentage (1,8-cineole; 0.6%).

### *Posidonia oceanica* (L.) Delile headspace VOCs

*Posidonia oceanica* (L.) Delile represents one of the most widespread species endemic to the Mediterranean Sea. Previous phytochemical studies showed that the mature leaves of *P*. *oceanica* are rich in amino acids, chicoric acid, *p*-coumaric acid, vanillin, ferulic acid, gentisic acid, caffeic and cinnamic acids, sterols and phenolic derivatives [41]. In chloroformic extract from *P*. *oceanica* a novel methylated sesquiterpene was identified (2*E*)-3,7,12-trimethyltridec-2-en-1-ol named posidozinol along with β-sitosterol and four fatty acids (palmitic, palmitoleic, oleic and linoleic) [42]. The occurrence of dimethylsulfoniopropionate (DMSP) and its variability during the seasonal cycle in the leaves of *P*. *oceanica* was reported [43]. However, there is no report on its headspace VOCs.

The main compound of *P*. *oceanica* headspace was DMS (59.3%; Table 1) that was expected since its precursor DMSP was previously identified in the leaves of this seagrass [43]. DMSP has been proposed to exhibit physiological roles (e.g. as an intracellular osmolyte and antioxidant), and also serves as a chemoattractant [26,44]. DMS (mediated by both bacterial and algal DMSP lyases) has been generated in oceans at remarkably high amounts (>107 tons per year) and is a key component of the ocean sulfur cycle and has a global role in atmosphere-ocean feedback processes [45,46]. Chlorophyceae, especially *Ulva*, *Enteromorpha*, and *Codium*, and red alga, *Polysiphonia*, are capable of producing large amounts of DMS in contrast to Phaeophyceae (brown algae) which produce little [47,48] that is in agreement with present research (Table 1). Namely, DMS percentage was the highest in *P*. *oceanica*, than in green alga (*F*. *petiolata*) and the lowest in brown algae (*Cystoseira* sp. and *D*. *linearis*). Besides higher alkanes pentadecane (14.5%) and heptadecane (7.2%) that were abundant in *F*. *petiolata* and *Cystoseira* spp. (Table 1), nonadecane was also found (6.3%).

### *Dictyota dichotoma* (Hudson) J. V. Lamouroux headspace VOCs

The genus Dictyota is represented by more than 40 species, thus being the richest genus of the family Dictyotaceae. In the Mediterranean in general, only the species *Dictyota dichotoma* and *Dictyota linearis* are found [49]. Brown algae of the family Dictyotaceae produce a significant number of secondary metabolites, especially diterpenes with three types of carbon skeletons: xenicanes; dolabellanes and ‘‘extended sesquiterpenes”. Many members of the family produce cyclic diterpenes, unique in the structural variety of marine natural products [49]. Terpenoid metabolites were identified in crude extract of *D*. *dichotoma* [49], e.g.: bicyclosesquiphellandrene, germacrene D, dictyoxide, pachydictyol A, isopachydictyol A, axenol, acetyldictyolal, dictyol B acetate, isopachydictyolal, 10-acetoxy-18-hydroxy-2,7-dolabelladiene, dictyol E, fucosterol, acetoxycrenulide, hydroxyacetyldictyolal, isodictyohemiacetal, dictyol A, dictyol B, dictyol C, and hydroxycrenulide. In addition, *D*. *linearis* contains different natural compounds [49] such as: dictyoxide, isopachydictyol A, pachydictyol A, dictyodial, 18-hydroxy-2,7-dolabelladiene, neodictyolactone, acetylsanadaol, acetyldictyolal, 10-acetoxy-18-hydroxy-2,7-dolabelladiene, dictyol E, 10,18-dihydroxy-2,7-dolabelladiene and dictyol C. There is no report on *D*. *dichotoma* headspace VOCs.

In distinction form other investigated samples, the major identified headspace VOCs of *D*. *dichotoma* were sesquiterpenes. Predominant sesquiterpene was germacrene D (28.3%; Table 1) that was previously found in *D*. *dichotoma* [49]. Bicyclogermacrene (4.7%) was also present. Sesquiterpenoids bicyclosesquiphellandrene, germacrene D and axenol were previously isolated from *Taonia atomaria* [50]. In this research, an array of sesquiterpenes biosynthetically connected with germacrene D were found (Table 1). They probably derived from protonation of germacrene D resulting in cadinenyl, muurolenyl, and amorphenyl cations that further react to form final products. Cadinenyl type sesquiterpenes were abundant: δ-cadinene (8.3%), γ-cadinene (3.4%), β-cadinene (2.8%), and *trans*-cadina-1,4-diene (1.2%). Epizonarene was found with 4.3% and it can be formed directly from germacrene D or from other intermediate cadinenes. It has been proposed that cyclization of germacrene D or of its endocyclic double bond isomer could result in the formation of bourbonene and copaene skeletons. β-Bourbonene was identified with (5.1%) and α-copaene with lower percentage. As a consequence of the thermodynamically unfavourable process to *cis*-decalin systems, the muurolenes and amorphenes are usually formed in smaller quantities that is in agreement with current research since they were found with lower percentages (α-muurolene (2.2%), γ-muurolene (2.1%), and α-amorphene (3.5%)).

### Possible biosynthetic origin of identified VOCs

The formation of DMS [1] results from an enzymatic decomposition of dimethyl-β-propiothetin, a metabolite of methionine that is fairly widespread in marine plants. Formed dimethylsulfoniopropionate (DMSP), a tertiary sulfonium compound involved in osmoregulation in algae, is the precursor of DMS. Recently, the algal enzyme responsible for formation of DMS from DMSP has been identified and characterized in algae *Emiliania huxleyi* [26].

Marine macroalgae exhibit a high ability to fix halide ions and form a variety of halogenated secondary metabolites [1]. In present research only tribromomethane and 1-iodopentane were found. A halogenating enzyme, haloperoxidase [29] is considered to participate in their synthesis in the presence of halides and hydrogen peroxide. Among them bromoperoxidases were detected in seaweeds (e.g. from *Corallina pilulifera* or *Ascophyllum nodosum*). The enzyme produces CHBr_3_ by its reaction with ketoacids, halide ions and hydrogen peroxide [51].

Both in terrestrial and aquatic ecosystems, VOCs of primary producers are usually dominated by lipid degradation products, and the overall mechanism for their enzymatic release is identical as in terrestrial plants [11] and algae [13]. The enzyme cascade is initiated by activated phospholipase, followed by lipoxygenase and hydroperoxide lyase, which leads to VOCs liberation. However, the particular enzymes are highly species- and sometimes even strain-specific [13] that can explain large biodiversity of volatile lipid degradation products (e.g. carbonyl compounds, alcohols, hydrocarbons). Marine algae contain C_20_, C_22_ and C_18_ unsaturated fatty acids, and they can produce both plant (C_18_) and animal type (C_20_ and C_22_) fatty acid hydroperoxides. Short-chain aldehydes (e.g. C_6_, C_9_) and middle-chain aldehydes (e.g. C_10_) that were particularly present in *H*. *filicina and F*. *petiolata* are mainly formed from fatty acids (C_20_) in marine algae (*via* hydroperoxides), whereas they are formed from C_18_ fatty acids in higher plants [52,53]. For example, the formation of hexanal is proposed *via* linoleic acid cascade and arachidonic acid cascade through their hydroperoxides as intermediates by the lipoxygenase/fatty acid hydroperoxide lyase pathway. It could also be provided by oxidation from other polyunsaturated fatty acids, as well as heptanal [54]. Lower aliphatic alcohols may be formed by decomposition of secondary hydroperoxides of fatty acids by the reduction of the corresponding aldehydes [55]. Octanal and nonanal could originate from ω9 mono-unsaturated fatty acids (MUFAs) and also from ω6 PUFAs such as linoleic acid [55]. Following this general concept of lipid peroxidation, and subsequent oxidative cleavage of the carbon skeleton, the biosynthesis of C_11_- and C_8_-hydrocarbons could start from a single precursor (e.g. eicosapentaenoic acid). The polyunsaturated fatty acid substrate could be activated [56] either by 9-lipoxygenase or by 12-lipoxygenase, and resulting 9- or 12-hydroperoxides that cleave oxidatively to produce characteristic C_11_- and C_8_-hydrocarbons (e.g. fucoserratene). However, the origin of 3-methylbutanal is well known. It is obtained from amino acids, and more particularly from leucine, during Maillard reactions by Strecker degradation [16].

Terpenoids are enzymatically synthesized from acetyl-CoA (mevalonate (MVA) pathway) and/or pyruvate (deoxyxylulose-5-phosphate (DXP) pathway) *via* precursor 2-isopentenyl pyrophosphate (2-IPP) and its isomer 3-isopentenyl pyrophosphate (3-IPP). Regular monoterpenes derived from geranyl pyrophosphate (GPP), but are not widespread among marine algae. More often are sesquiterpenes derived metabolically from 300 distinct C_15_ -hydrocarbon skeletons, which in turn are produced from farnesyl diphosphate (FPP) by the action of sesquiterpene synthases [57]. The structure elucidation, biosynthesis, and biological activity of marine carbotricyclic sesquiterpene compounds were reviewed [10].

Carotenoid cleavage dioxygenases (CCDs) catalyze oxidative cleavage of carotenoids, resulting in the production of norisoprenoids—apocarotenoids [58] *via* glycosylation and breakdown of stored glycosides by glycosidase enzymes. CCDs often exhibit substrate promiscuity, which probably contributes to their natural diversity. Non-enzymatic reactions involving one or several steps of carotenoid degradation, stimulated by light, oxygen, temperature and acid hydrolysis can also occur [59]. The breakdown products of carotenoids found in this research belong to C_13_-norisoprenoids with megastigmane structure. In addition, C_11_-non-isoprenoids were identified. Dictyopterene A ((1*R*,2*S*)-*cis*-1-vinyl-2-(*trans*-1’-hexenyl)cyclopropane) and dictyoterpene B ((1*R*,2*S*)-*cis*-1-vinyl-2-(*trans*-1,*cis*-3-hexadienyl)cyclopropane) are proposed precursors [1] of dictyopterene C (6-butylcyclohepta-1,4-diene) and dictyopterene D (6-[(1*Z*)-but-1-enyl]cyclohepta-1,4-diene). Proposed biogenesis of dictyopterenes starts from (3*S*)-1,*cis*-5-undecadien-3-ol and (3S)-1,*cis*-5,*cis*-8-undecatrien-3-ol by dehydration and cyclization.

Benzenoid and phenylpropanoid volatile compounds, primarily derived from phenylalanine require shortening of the carbon skeleton side chain by a C_2_-unit, which can potentially occur *via* either the β-oxidative pathway or non-oxidatively [60].

## Conclusions

Considering limited data available on the chemical composition of marine plants from the Adriatic Sea, the present research is contribution toward their better chemical characterization. Significant differences were found among the headspace VOCs from 3 seaweeds and 1 seagrass. High abundance of DMS was found in *P*. *oceanica* followed by *F*. *petiolata* and *H*. *filicina* indicating those plants as source of sulfur compounds in marine ecosystem. Their headspace contained individually variety of C_8_-compounds (e.g. fucoserratene), benzaldehyde, alkanes C_15_, C_17_ and C_19_, dictyopterene D and C, others. Sesquiterpenes were found in *D*. *dichotoma*, predominantly germacrene D indicating similarity to terrestrial aromatic plants. Identified VOCs contain different types of organic compounds that may be significant for chemosystematics and ecology (chemical communications in marine ecosystems).
